# The cervicovaginal gut microbiota axis as a key determinant of systemic physiology

**DOI:** 10.1080/19490976.2026.2726648

**Published:** 2026-09-21

**Authors:** Eric H. Rosenn, Maayan Zuniga, Maya Lipshitz, Eryn Fox, Max Engel, Giulio Maria Pasinetti

**Affiliations:** a Department of Biomedical Engineering, New York University, Tandon School of Engineering, New York, NY, USA; b School of Medicine, Tel Aviv University, Tel Aviv, Israel; c College of Osteopathic Medicine, Touro University, New York, NY, USA; d Department of Allergy and Immunology, Montefiore Medical Center, Albert Einstein College of Medicine, New York, NY, USA; e Department of Neurology, Icahn School of Medicine at Mount Sinai, New York, NY, USA; f Geriatric Research, Education and Clinical Center, James J. Peters Veterans Affairs Medical Center, Bronx, New York, USA

**Keywords:** Microbiome, gut-brain axis, dysbiosis, multi-omics, immunometabolism, cervicovaginal-gut axis, cervicovaginal microbiome

## Abstract

The gut and vaginal microbiomes are both essential to human health, yet they have largely been studied in isolation. Gut microbiota are central to host metabolism, immunity, and the gut–brain axis, while the vaginal microbiome, typically dominated by *Lactobacillus* species, protects against infection and shapes reproductive outcomes. Although dysbiosis at either site has been linked to systemic disease, the possibility that these communities are interrelated and that the vaginal microbiome could serve as a clinically accessible proxy for systemic microbial states remains underexplored. This represents a clear gap in the literature. In this review, we examine physiological connections between digestive and genitourinary systems, explore how endocrine and immune networks exert dual control across compartments, and analyze correlations between dysbiosis and disease states. We then consider emerging technologies for microbiome analysis and their clinical and translational implications, including the potential for vaginal microbiome profiling to function as a diagnostic surrogate for the gut and as a complementary biomarker platform for prediction of neurologic disease risk. By building a systems-level understanding of the joint immuno-microbial interactome, evaluating advances in computational and omics-based technologies, and situating these tools within emerging healthcare frameworks, we argue that the cervicovaginal-gut microbiota axis is a unique and understudied source for medical discovery.

## 1. Introduction

The human microbiota refers to the collection of microorganisms, including bacteria, viruses, fungi, and archaea, inhabiting distinct niches such as the gut, oral cavity, skin, and genitourinary system.^1^ Throughout this review, the term **microbiome** is used when referring to these microorganisms together with their collective genomes, metabolites, and surrounding microenvironment.^2^ While the gut microbiome has received substantial attention for its role in metabolic homeostasis, immune regulation, and neuroendocrine signaling,^3^ emerging evidence highlights the equally critical influence of the vaginal microbiome on systemic physiology, reproductive outcomes, and disease susceptibility.^4^ ^,^ ^5^ The gut and genital tract microbiota constitute dynamic ecosystems that are integral to host physiology and disease resistance.^6^ The gut harbors an estimated 10^13^-10^14^ microorganisms, making it one of the most densely populated microbial communities in the body.^7^ By contrast, the upper reproductive tract is only intermittently colonized, whereas the cervicovaginal niche harbors billions of microbes that maintain mucosal integrity and protect against infection.^8^ ^,^ ^9^

Host–microbe interactions span commensal, mutualistic, and parasitic relationships.^10^ Mutualism is exemplified by gut microbes performing anaerobic fermentation of dietary polysaccharides. These processes release secondary metabolites such as short-chain fatty acids (SCFAs) that provide energy to colonocytes, reinforce epithelial barriers, and calibrate immune responses.^11^ In contrast, *Giardia* infection can disrupt gut microbial networks, compromise epithelial function, and cause diarrheal disease.^12^

The vaginal microbiota exhibits similar ecological dynamics. Dominance by *Lactobacillus* species promotes resilience through lactic acid production, maintenance of acidic pH, and secretion of bacteriocins and hydrogen peroxide.^13^ ^,^ ^14^ Dysbiosis, characterized by depletion of protective taxa and overgrowth of anaerobes, constitutes a critical risk state that increases susceptibility to bacterial vaginosis (BV), facilitates sexually transmitted infection (STI) acquisition, and jeopardizes reproductive health.^15-17^

Dietary intake influences both gut and vaginal microbial communities by modulating hormone levels, nutrient availability, and microbial metabolism.^18^ ^,^ ^19^ In the vagina, estrogen-driven glycogen deposition is essential for sustaining *Lactobacillus* dominance.^20^ Two dietary hypotheses have been proposed to explain the phenomenon: first, high-starch diets may raise vaginal glycogen and thereby favor *Lactobacillus*, and second, high-fat diets may increase circulating estradiol, indirectly supporting *Lactobacillus* growth.^21-23^

The gut microbiome responds strongly to macronutrients such as protein, fat, sugar, starch, and fiber, which may in turn influence the vaginal niche.^24^ The gastrointestinal tract has been proposed as a reservoir for vaginal taxa, with gut colonization by either *Lactobacillus* or BV-associated bacteria correlating with BV risk.^25^ Direct perineal transfer between the rectum and vagina provides a plausible route of microbial exchange.^26^ Furthermore, epidemiologic studies using Nugent scoring have linked high dietary fat and low micronutrient intake to increased BV prevalence, suggesting that diet may shape vaginal health both directly and indirectly.^27^ ^,^ ^28^

Cross-system regulation also occurs through endocrine and metabolic mechanisms such as estrogen metabolism. The gut microbiome modifies estrogen via *β*-glucuronidase activity,^29^ permitting deconjugation and reabsorption through enterohepatic recycling and thereby influencing systemic estrogen availability.^30^ Alterations in this pathway have been associated with estrogen-related disorders of the reproductive tract, including endometrial cancer, endometriosis, uterine fibroids, and polycystic ovary syndrome (PCOS).^31^ Collectively, these insights have brought into focus the concept of a gut–vaginal axis, a framework linking diet, microbial metabolites, endocrine networks, and mucosal immunity in the coordination of gastrointestinal and reproductive health.^15^

Evidence supporting links between the gut and cervicovaginal microbiomes spans multiple levels, including epidemiologic associations, shared endocrine regulation, immune signaling, and metabolite-mediated communication.^32^ Across studies, microbial metabolites such as SCFAs, bile acid derivatives, and tryptophan catabolites, together with estrogen metabolism, provide plausible mechanisms by which host physiology can shape microbial niches across body sites in parallel.^33^ ^,^ ^34^ At the same time, longitudinal human studies that concurrently profile gut and cervicovaginal microbiomes across pregnancy remain scarce, limiting the ability to assess coordinated inter-site microbial dynamics during gestation.^35^ Recent reviews in the context of cervicovaginal immunity, human papillomavirus (HPV)-related disease, and cervical carcinogenesis further emphasize how vaginal community structure can modulate mucosal immune tone and disease susceptibility, reinforcing the systemic relevance of microbiome-linked pathways.^36-38^

This review explores the physiological foundations of this axis, synthesizes evidence for cross-compartment dysbiosis, and highlights its translational implications for diagnostics, disease prevention, and microbiome-targeted interventions. The strength of evidence supporting this axis varies across mechanisms. Human studies support shared endocrine, immune, and metabolic regulation and, in selected populations, overlap between rectal and vaginal taxa. However, explicit directional microbial exchange, consistent cross-site concordance, and use of vaginal profiles as surrogates for gut or systemic states remain incompletely established and are treated here as hypothesis-generating translational avenues.^39^ ^,^ ^40^

### 1.1. Intro to the gut microbiome

**Figure 1.**
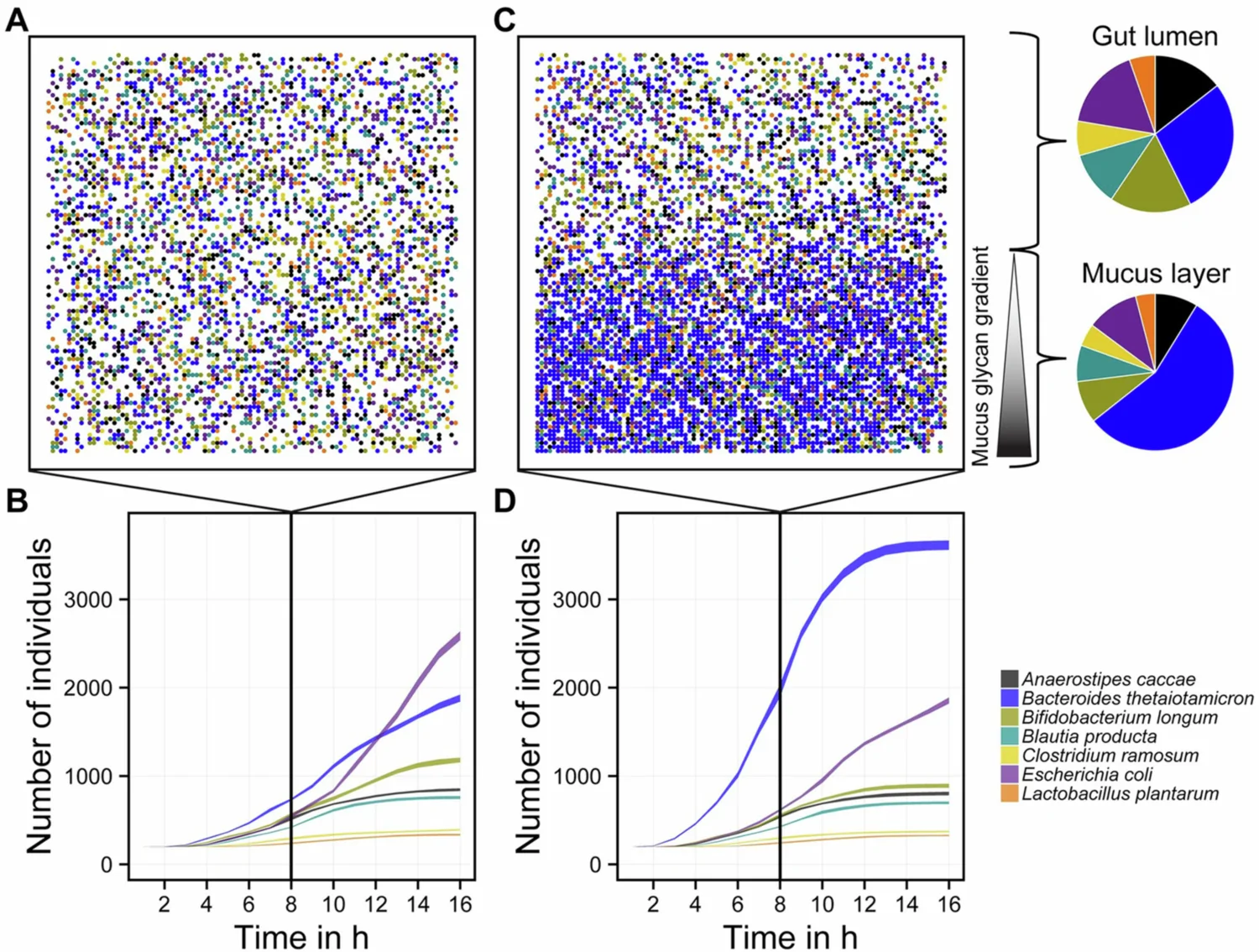
Spatial dynamics of a representative gut microbiome community. Bauer et al. ^45^ used BacArena to simulate the growth and spatial dynamics of a representative gut microbial community. In one scenario, species were cultured for 8 hours in a rich medium with nutrients uniformly distributed, producing characteristic growth curves for each taxon (A, B). In a second scenario, the same community was simulated under a spatial gradient of mucus glycans, which led to distinct layering of species between the gut lumen and mucus layer and corresponding shifts in growth trajectories (C, D). These simulations demonstrate how gut flora can be modeled as structured, dynamic ecosystems rather than static species inventories.

Tens of trillions of microorganisms inhabit the average human gastrointestinal tract, including diverse species of bacteria, archaea, fungi, and viruses, with the highest density in the colon and distal ileum.^41^ *Firmicutes* and *Bacteroidetes* account for approximately 90% of gut bacteria by relative abundance, with Actinobacteria, Proteobacteria, Fusobacteria, and Verrucomicrobia present in smaller proportions. Microbial classification into **enterotypes** is based on the relative abundance of dominant bacterial genera, most commonly *Bacteroides*, *Prevotella*, and *Ruminococcus*.^42^ ^,^ ^43^ Each enterotype represents a stable community pattern defined by proportional species abundance and functional outputs (Figure 1).^44^

To give a brief anatomical context: the intestinal epithelium forms the primary barrier between the microbiota and host tissues. It is composed of a single layer of intestinal epithelial cells (IECs) sealed by tight junction proteins (claudins, occludin, zonula occludens).^46^ A mucus layer rich in MUC2 mucin covers the epithelium, providing both a physical barrier and substrate for commensals.^47^ ^,^ ^48^ Antimicrobial peptides (defensins, Reg III *γ*, cathelicidins) are secreted by Paneth cells, while IgA, produced by plasma cells in Peyer’s patches, coats microbes to promote immune exclusion.^49^ The gut-associated lymphoid tissue (GALT), comprising Peyer’s patches and mesenteric lymph nodes, orchestrates adaptive immune responses and links the gut to the broader common mucosal immune system.^50^ ^,^ ^51^

Gut microbiota generate metabolites from three main sources: (1) host-synthesized substrates modified by microbes (secondary bile acids), (2) microbial transformations of dietary substrates (SCFAs, indoles), and (3) *de novo* microbial synthesis (vitamins, polysaccharide A, neurotransmitters).^52^ ^,^ ^53^ These metabolites regulate gut motility, barrier integrity, host metabolism, immune responses, and systemic physiology, including circadian rhythms and drug metabolism.^50^

SCFAs specifically, including acetate, propionate, and butyrate, are produced by fermentation of resistant starches, inulin, and other nondigestible polysaccharides by *Firmicutes*, *Bacteroidetes*, and *Actinobacteria* ^11^. It is well established that the byproducts of microbial metabolism are essential for human health. Butyrate, for instance, is the primary energy source for colonocytes and maintains epithelial tight junctions. Propionate supports hepatic gluconeogenesis and cholesterol metabolism,^54^ ^,^ ^55^ while acetate enters peripheral circulation and contributes to lipid and appetite regulation.^56^ SCFAs also play a role in intestinal homeostasis by acidifying the colonic lumen, suppressing pathogens such as *C. difficile*, *C. perfringens*, *Campylobacter*, *E. coli*, *S. aureus*, and *Yersinia*, while favoring *Bifidobacterium* and *Lactobacillus*.^57^ ^,^ ^58^ While much evidence has been gathered regarding the clinical implications of these metabolic networks, therapeutics leveraging the microbiome remain largely in basic and translational stages of research.^59^

SCFAs are also involved in immunometabolic signaling; absorbed by diffusion or active transporters such as MCT1 and SMCT1, they stimulate G-protein-coupled receptors including FFAR2 (GPR43), FFAR3 (GPR41), and HCAR2 (GPR109A).^60^ These receptors are broadly expressed across intestinal epithelial, adipose, and immune cells.^61^ As receptor signaling molecules, butyrate and propionate inhibit histone deacetylases, reprogramming transcriptional profiles of T-cells and dendritic cells. Collectively, SCFAs attenuate proinflammatory cytokines (TNF-*α*, IFN-*γ*, IL-6) ^62^ ^,^ ^63^ and promote anti-inflammatory pathways, including IL-10 secretion and Treg-cell expansion.^64^

Tryptophan catabolism by microorganisms produces indole derivatives such as indole-3-acetic acid and indole-3-aldehyde. These ligands act on the aryl hydrocarbon receptor (AhR), which plays a role in modulating epithelial integrity and mucosal innate immunity.^65^ Secondary bile acids, produced by microbial deconjugation and dehydroxylation, are essential for lipid absorption and modulate hepatic immune pathways through FXR.^66^ These metabolites also activate TGR5, a G-protein–coupled bile acid receptor expressed on intestinal epithelial cells, monocytes, and enteric neurons, highlighting a key intersection of microbial immunometabolism with neuroendocrine signaling.^67^ Gases produced by microbes, such as hydrogen, methane, and hydrogen sulfide, have a broader impact on gastric motility and redox conditions.^68^ ^,^ ^69^ However, the compounds produced by gut microbes with still more well-known health implications include vitamins (K, B12, folate, riboflavin) and neurotransmitters (GABA, serotonin precursors, dopamine metabolites).^5^ ^,^ ^53^ ^,^ ^70^

Eubiosis refers to a state where microbial species at microbiome locations such as the mouth, skin, gut, and urinary tract have healthy, stable population densities, associated with local homeostasis. In the gut, healthy *Lactobacillus* and *Bifidobacterium* levels enhance mucus secretion, stimulate antimicrobial peptides, promote IgA production, and prevent harmful pathogen adhesion.^47^ Dysbiosis refers to the disruption of community structure or function, often defined as an enterotype diverging from a health-associated configuration.^71^ ^,^ ^72^ Dysbiosis is characterized by a loss of diversity, depletion of beneficial taxa, and overgrowth of opportunists.^73^ Functional consequences include reduced SCFA and indole production, weakened tight junctions, increased gut permeability, and systemic translocation of bacterial products such as lipopolysaccharide (LPS), which activates TLR4 signaling and drives systemic inflammation.^74-76^

Dysbiosis contributes to gastrointestinal disorders such as inflammatory bowel disease (IBD) and irritable bowel syndrome (IBS); metabolic and cardiovascular disease, including obesity, diabetes, and atherosclerosis; and extraintestinal diseases involving the central nervous system, including Parkinson’s disease, Alzheimer’s disease, schizophrenia, and major depressive disorder.^77-79^ Enrichment of *Enterobacteriaceae* and *Streptococcus* is more broadly linked to vascular inflammation and endothelial dysfunction.^80^ ^,^ ^81^

### 1.2. Intro to the cervico-vaginal microbiota

The cervicovaginal microbiome functions as both a barrier and a biosensor, linking microbial ecology with mucosal immunity and reproductive health. Dysbiosis disrupts microbial metabolite profiles, induces inflammation, compromises epithelial and immune barriers, and increases susceptibility to STIs and reproductive tract disease.^82^ ^,^ ^83^

**Figure 2.**
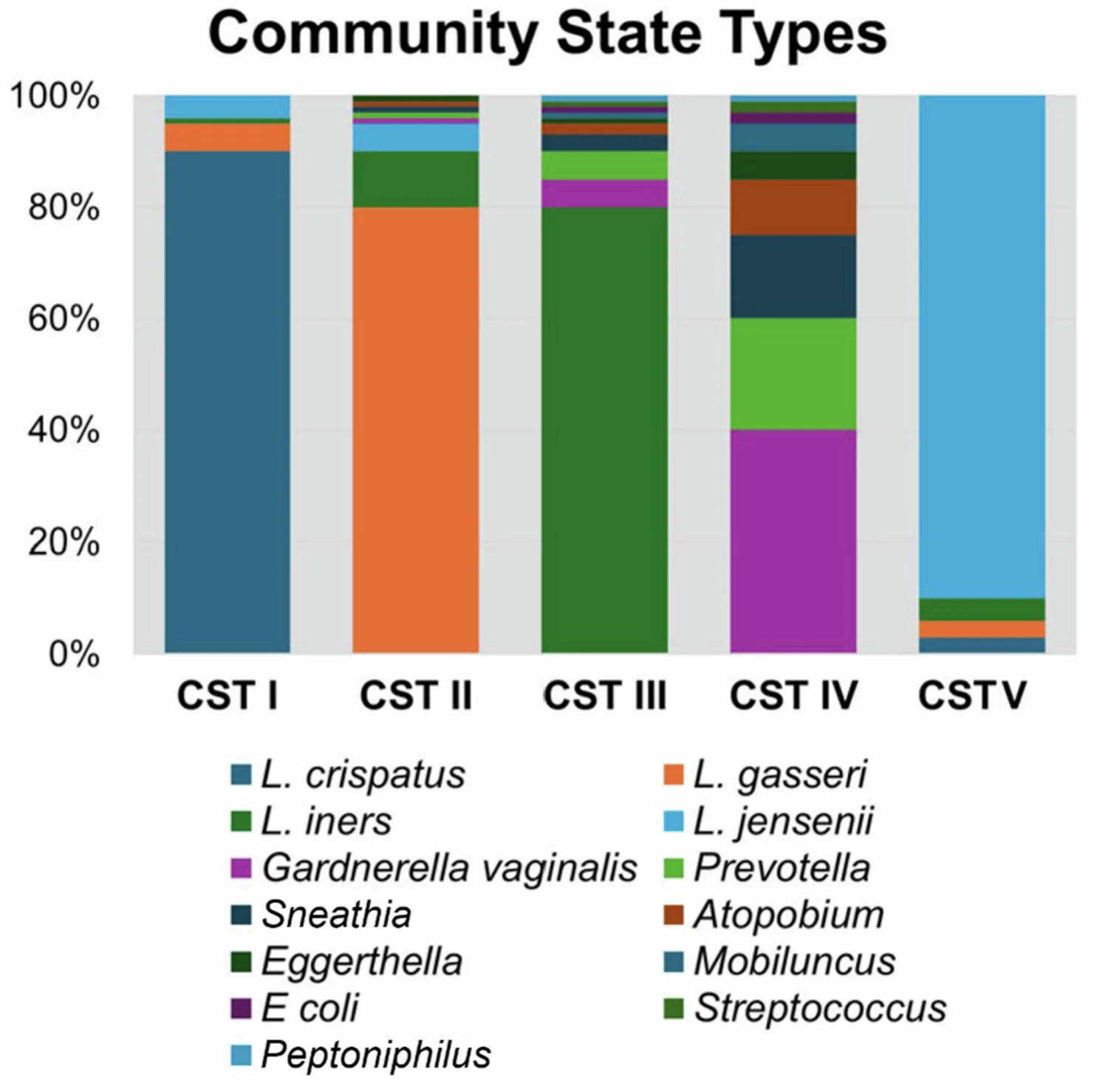
Vaginal Microbiome graphs representing relative abundance of taxa within each CST group. Adapted from Orton and Monaco.^92^ Vaginal enterotypes are classified into five CSTs that represent the most common biome distributions among reproductive-age women. Among CSTs defined by Ravel et al. ^9^, four are dominated by distinct Lactobacillus species (*L. crispatus, L. gasseri, L. iners, and L. jensenii*), whereas CST-IV represents a heterogeneous anaerobic consortium, frequently enriched with *Gardnerella, Prevotella,* and *Atopobium* (more modernly termed *Fannyhessea*). Although most women exhibit *Lactobacillus* dominance, approximately one quarter of women are stably colonized by CST-IV communities despite being asymptomatic, highlighting the complexity of defining ‘healthy’ vaginal states.^93^ CST distribution varies by ethnicity, with a higher prevalence of CST-IV among women of African ancestry.^94^ ^,^ ^95^

To provide brief anatomical context, the vagina and ectocervix are lined by stratified squamous epithelium, while the endocervix is lined by simple columnar epithelium. The transformation zone, where squamous metaplasia occurs, represents a dynamic epithelial interface with heightened immune activity and the greatest susceptibility to HPV-related carcinogenesis.^84-86^ Cervical mucus contains mucins (MUC1, MUC4, MUC5AC, MUC5B, MUC6), antimicrobial peptides (defensins, lysozyme, secretory leukocyte protease inhibitor), and immunoglobulins (secretory IgA, IgG), providing both physical and chemical barriers.^87^ ^,^ ^88^ Lactobacilli metabolize epithelial glycogen–derived sugars such as glucose and maltose to produce lactic acid, sustaining a vaginal pH of 3.8–4.2 that restricts pathogen growth.^89^ ^,^ ^90^ A description of five community state types (CSTs) used for enterotype classification can be found in Figure 2. Here, enterotype is used in the broad sense of a microbial composition defined by the relative abundance of constituent taxa. In the cervicovaginal microbiome, these compositions are more commonly described as community state types (CSTs), which represent recurring stable community configurations observed across populations. Although individual equilibria vary, most profiles cluster into a limited number of characteristic CST distributions.^91^

The cervicovaginal mucosa represents a uniquely immune-active interface, balancing tolerance of commensals, sperm, and the semi-allogeneic fetus with rapid responses to invading pathogens.^96^ Dendritic cells and macrophages, distributed throughout the epithelium and submucosa, continuously sample microbial antigens and shape adaptive immune responses.^97^ This environment is enriched in effector memory T cells, reflecting persistent antigenic exposure and conferring rapid recall capacity.^98^ Notably, CD8⁺ tissue-resident memory T cells (TRMs), defined by CD69 and CD103 expression, are particularly abundant and encompass functionally distinct subsets with antiviral, tissue-protective, and barrier-stabilizing roles.^99^ Their strategic localization enables immediate containment of viral infections such as human immunodeficiency virus (HIV) and HPV, and their presence has implications for vaccine efficacy and mucosal immunotherapy.^100^

Innate immune cells provide the first line of cervicovaginal defense. Neutrophils respond rapidly to microbial invasion and tissue injury, whereas natural killer (NK) cells exert antiviral effects through cytotoxicity and cytokine secretion.^101^ Antigen-presenting cells express pattern recognition receptors, including TLR2 (bacterial lipoproteins), TLR4 (lipopolysaccharides), and TLR9 (bacterial and viral DNA), thereby linking microbial sensing to adaptive immune activation.^102^ ^,^ ^103^ Humoral protection is mediated by secretory IgA, which coats microbes and limits epithelial adherence, and by IgG, derived largely from systemic circulation, which bridges systemic and mucosal immunity.^104^ ^,^ ^105^ Together, these layered defenses establish a highly specialized immune environment that is protective against pathogens but can be disrupted during dysbiosis or exploited by STIs.^106^

The vaginal microbiome is a dynamic ecosystem that shifts in response to hormonal changes and life stage, with important consequences for reproductive health.^107^ In childhood, low estrogen levels support a neutral pH and sparse *Lactobacillus* colonization.^108^ Puberty initiates a major ecological transition: rising estrogen drives glycogen deposition in the epithelium, fueling *Lactobacillus* expansion and acidification of the vaginal environment. During menstruation, the presence of blood temporarily elevates pH and reduces *Lactobacillus* abundance,^109^ whereas pregnancy stabilizes communities by favoring *Lactobacillus* dominance and reducing overall diversity.^93^ ^,^ ^110^ With menopause, estrogen decline reverses these trends, leading to diminished *Lactobacillus* prevalence and increased vaginal pH.^111^ ^,^ ^112^ Together, these endogenous and exogenous forces shape the cervicovaginal microbiome across the female lifespan.^113^

Advanced three-dimensional cervical epithelial models have provided critical insights into how commensal and dysbiotic bacteria differentially shape mucosal responses.^114^ *Lactobacillus crispatus* preserves epithelial barrier integrity and maintains immune quiescence without eliciting proinflammatory cytokines.^115-117^ By contrast, species associated with dysbiosis such as *Gardnerella vaginalis* and *Prevotella bivia* induce modest inflammatory responses, characterized by the production of IL-6, TNF-*α*, and IL-1α, often accompanied by matrix metalloproteinase-9 (MMP-9) activation.^118-121^ More virulent taxa, including *Fannyhessea vaginae* and *Sneathia amnii*, provoke a broader inflammatory cascade encompassing cytokines, chemokines, and matrix-remodeling enzymes that destabilize epithelial architecture.^122^ These microbe-specific immune signatures elucidate the mechanisms by which dysbiosis compromises barrier function, amplifies mucosal inflammation, and heightens susceptibility to sexually transmitted pathogens.^106^ ^,^ ^123-125^

Microbial metabolites are central mediators of host–microbe interactions at the cervicovaginal surface.^126^ Under *Lactobacillus* dominance, abundant lactic acid suppresses TLR-driven proinflammatory cytokines, increases the IL-1 receptor antagonist, and dampens IL-6 and IL-8 responses.^127^ *L. gasseri*–derived exopolysaccharides further bias immunity toward tolerance by enhancing IL-10 and reducing TNF-*α*.^128^ This metabolic profile, characterized by high lactic acid, low SCFAs, and a pH of ~3.9, reinforces barrier function and limits inflammation.^129^

Dysbiosis, by contrast, generates a metabolite profile dominated by acetate, succinate, and butyrate at a higher pH (~7), conditions that fuel mucosal inflammation.^34^ ^,^ ^130^ ^,^ ^131^ BV-associated taxa also produce oxidative intermediates such as 2-hydroxyglutarate and 2-hydroxybutyrate, linked to reactive oxygen species, lipid peroxidation, and barrier breakdown.^34^ ^,^ ^132^ ^,^ ^133^ Beyond metabolic disruption, dysbiotic communities perturb host microRNA expression and drive epithelial apoptosis, necrosis, and permeability defects.^134^ Together, these changes shift the cervicovaginal environment from a state of immune quiescence and barrier integrity to one characterized by chronic inflammation and heightened susceptibility to infection.^135-137^ Recent tumor-microbiome evidence further supports a local metabolite-mediated mechanism, with a tumor-resident bacterium reported to promote cervical cancer progression through lysoPC-mediated activation of c-Jun/c-Fos signaling.^138^

Community State Type IV (CST-IV) and BV exemplify unstable vaginal community structures that heighten susceptibility to STIs including HIV and HPV.^139^ ^,^ ^140^ Persistent CST-IV states in particular are linked with HPV acquisition, persistence, and progression to cervical neoplasia.^141-143^ Persistent HPV infection and host immune evasion provide the mechanistic oncogenic context in which dysbiotic cervicovaginal communities may influence cervical carcinogenesis.^38^ In cervical cancer, the vaginal microbial environment has also been reported to skew macrophage polarization, linking dysbiosis-associated immune remodeling with tumor-permissive inflammatory programs.^82^ Consistent with this organism-specific framework, *Peptostreptococcus anaerobius* has been reported to promote cervical cancer angiogenesis through SCD upregulation and ERK pathway activation.^144^ Recent synthesis of the cervical cancer literature further highlights vaginal microbiome-immune crosstalk as a potential contributor to tumor immune modulation and microbiome-informed prevention or therapeutic strategies.^36^

Dysbiosis extends beyond infection risk, contributing to pelvic inflammatory disease, infertility, and adverse pregnancy outcomes including preterm birth.^111^ ^,^ ^145^ Transitional dominance by *Lactobacillus iners*, often in conjunction with *Gardnerella vaginalis*, further reflects ecological instability and correlates with recurrent dysbiosis.^146^ ^,^ ^147^ By contrast, *Lactobacillus*-dominated communities establish a protective state characterized by lactic acid production, acidic pH, epithelial barrier reinforcement, and balanced mucosal immunity.^14^ ^,^ ^148^ Dysbiosis, in turn, disrupts this equilibrium: altering metabolite profiles, amplifying proinflammatory cytokines, and weakening barriers, thereby fostering pathogen overgrowth and reproductive tract disease. Recognizing these opposing ecological states provides a framework for developing microbiome-based diagnostics and interventions to improve women’s reproductive health.^149^ ^,^ ^150^

## 2. Evidence for a gut-vaginal microbiota axis

This section explores how the gut and vaginal microbiomes are functionally connected through a discussion of the physiological mechanisms that facilitate cross-talk and the roles of endocrine and immune networks in coordinating both niches. In disease states, parallel disturbances across compartments further emphasize shared regulatory pathways. Collectively, this body of evidence supports the biological plausibility and clinical relevance of a gut-vaginal axis, while also indicating that the strength and directness of evidence vary by mechanism, disease context, and study design.

Two distinct forms of coupling are considered. Section 2.1 examines routes through which gut-derived organisms, metabolites, immune mediators, or microbial hormone metabolism may influence the genitourinary environment. Section 2.2 examines common-driver coupling, in which systemic endocrine signals independently reshape both microbiomes and may therefore generate correlated or potentially predictive patterns without requiring direct inter-compartment transfer.

### 2.1. Physiologic routes of gut-to-genitourinary signaling and cross-site exchange

The gut microbiota not only colonizes the gastrointestinal tract but also influences the composition and resilience of the cervicovaginal and urinary tract microbiomes. Research over the past decade has identified several pathways through which this connection manifests, including endocrine regulation, immunologic signaling, microbial translocation, and metabolite circulation.^151^ ^,^ ^152^

A major avenue of interaction involves hormonal metabolism. Table 1 summarizes hormone-microbiome interactions observed across experimental systems, including non-human models; these studies are included to highlight conserved endocrine and microbial mechanisms that inform hypotheses in basic research. The gut microbiome functions as a quasi-endocrine modulator through its capacity to metabolize sex steroids. Bacterial *β*-glucuronidases and sulfatases deconjugate estrogens excreted in bile, permitting their reabsorption and recirculation in the enterohepatic system.^153-155^ This pathway can influence the cervicovaginal environment, as systemic estrogen availability regulates epithelial glycogen accumulation and supports *Lactobacillus*-associated acidification.^31^ ^,^ ^155^ Clinical and observational studies report that disruptions in gut microbial balance are associated with altered systemic estrogen levels and with conditions such as endometriosis,^156^ PCOS,^157^ and postmenopausal vaginal atrophy.^158^ ^,^ ^159^ These findings establish a hormonal pathway through which gut microbial dynamics shape genitourinary health.

**Table 1.** Gut microbes related to sex hormone synthesis.

Hormone	Model	Finding	Microbiota studied	Ref.
Insulin	Honey bees	Changes in insulin signaling reduces gut pH and gustatory response	*Gilliamella apicola* and *Lactobacillus spp*.	[160]
Testosterone, estradiol	Human	Correlation of diversity of microbiota with sex hormones	*Acinetobacter, Dorea, Ruminococcus, and Megamonas, Bacteroidetes* and *Firmicutes*	[161]
Adrenocorticotropic	Wistar rats	Role of gut microbiota in depression	*Desulfovibrionales, Desulfovibrio, Klebsiella, Burkholderiales,* and *Bifidobacterium*	[162]
Ghrelin	Wistar rats	Short-chain acid-producing bacteria and its effects on gut microbiota	*Turicibacter, Brevibacterium, Parasutterella,* and *Oscillibacter*	[163]
Juvenile hormone III	Riptortus pedestris	A more significant number of eggs produced as a result of *Burkholderia* gut symbiont	*Burkholderia*	[164]
Adrenocorticotrophic hormone	Wistar rats	Changes in the microbiome diversity	*Akkermansia* and *Lactobacillus*	[165]
Epinephrine and Norepinephrine	Bacterial growth	Stress hormones can affect the growth of anaerobic bacteria in the gut	*Fusobacterium nucleatum, Prevotella, Porphyromonas spp., Tannerella forsythia*, and *Propionibacterium acnes*	[166]
Thyroxine	Mice	Reduced diversity of some bacterial species	Bacteria belonging to families *Lactobacillaceae* and *Bifidobacteriaceae*	[167]
Androgen	Pigs	Influence of host gender on gut microbiota and sex-biased bacterial taxa	Bacteria belonging to families *Veillonellaceae, Roseburia, Bulleidia,* and *Escherichia*	[168]
Androgen	Wistar rats	Prenatal androgen was associated with the abundance of gut microbiota	*Akkermansia, Bacteroides, Lactobacillus, Clostridium, Nocardiaceae,* and *Clostridiaceae*	[169]
Glucagon-like peptide 1	Mice	Intestinal microbiota increases GLP-1 levels	*L. paracasei, L. bulgaricus,* and *Streptococcus thermophilus*	[170]
Corticosterone, adrenocorticosterone	Probiotics in rats and humans	Bacteria reduce levels of stress hormones	*L. helveticus* and *B. longum*	[171]

The intestinal and vaginal mucosae are part of the common mucosal immune network.^172^ ^,^ ^173^ Antigen exposure in one mucosal site may influence immune reactivity at distant sites through the trafficking of lymphocytes and dendritic cells, as demonstrated primarily in animal models.^174^ During dysbiosis, increased intestinal permeability allows microbial products, including lipopolysaccharides and peptidoglycan fragments, to enter the circulation, thereby triggering systemic inflammatory cascades.^175^ Elevated systemic inflammation has been associated with reduced Lactobacillus dominance in the vaginal microbiome; thus, predisposing to BV, recurrent urinary tract infections, and adverse reproductive outcomes.^16^ ^,^ ^176^ ^,^ ^177^ Conversely, chronic cervicovaginal inflammation, such as that observed in persistent high-risk HPV infection, has been shown to affect systemic cytokine levels and metabolic regulation, suggesting a reciprocal influence on gut immune tone.^17^ ^,^ ^178-180^ Dysbiosis is associated with increased genital IL-1β concentrations and increased neutrophils or potentially other leukocytes.^181^ ^,^ ^182^ It is hypothesized that certain pathogenic bacteria contribute to BV by inhibiting TLR activation by downregulating host heat shock protein production, processes that collectively induce a proinflammatory response.^182^ In addition to influencing the secretion of cytokines, lactobacilli in the vagina produce antimicrobial molecules that stimulate the production of IL-8, IL-6, IL-1β, and TNF-*α*, in response to pathogen challenge.^182-184^

Anatomical proximity and paired rectal-vaginal studies support the rectum as a reservoir for selected vaginal and urogenital taxa.^185-188^ Identical genotypes of several bacterial species have been recovered from rectal and vaginal samples from the same patient.^185^ ^,^ ^189^ In a prospective cohort, baseline detection of BV-associated bacteria in oral or anal samples was associated with subsequent BV acquisition, suggesting that pre-existing extravaginal reservoirs may precede incident vaginal dysbiosis.^190^ Recent paired shotgun-metagenomic analyses have also identified community patterns consistent with rectal-to-genital and genital-to-rectal microbial sharing,^191^ ^,^ ^192^ while recurrent urinary tract infection studies provide adjacent evidence for rectal, vaginal, and urinary reservoir communication.^193^ ^,^ ^194^ However, simultaneous detection at both sites does not establish directionality, and strain-resolved pregnancy studies have sometimes identified genetically divergent strains despite species-level overlap.^195^ Collectively, these findings support cross-site seeding of selected organisms but do not establish that direct gut-to-vaginal transfer is common across the broader microbiome.

Systemic antimicrobial exposure can disrupt non-target host-associated microbiota, and recent entero-vaginal dysbiosis modeling supports the concept that antibiotic disruption can affect intestinal and vaginal ecosystems in parallel.^196^ In humans, azithromycin treatment for genital *Chlamydia trachomatis* infection has been associated with persistent shifts in vaginal community structure, including enrichment of *Lactobacillus iners*-dominated communities after treatment.^197^ High-resolution longitudinal profiling during oral metronidazole therapy for bacterial vaginosis further shows rapid but variable vaginal microbial and immune remodeling during and after treatment.^198^ Longitudinal stool metagenomics after antibiotic exposure also demonstrate gut microbial, functional, phageome, and antimicrobial-resistance perturbations with incomplete or variable recovery, but these gut-focused studies do not establish that gut perturbation consistently precedes or directly causes subsequent vaginal change.^199^

Li et al. propose that metabolites released into circulation by certain gut microbes may help regulate the vaginal microbiome.^200^ Notably, 3-phosphoglycerate and lysophosphatidylcholine were identified as potential antagonistic mediators of vaginitis-associated effects involving gut microbial taxa, while *Gardnerella vaginalis* and *Prevotella bivia* remain key BV-associated organisms within dysbiotic vaginal communities.^183^ ^,^ ^201^ SCFAs generated by fermentation of dietary fiber in the colon connect the gut microbiome with mucosal and systemic immune signaling, including antiviral immune responses.^202-204^ In healthy adults, lactate, succinate, and 1,2-propanediol are typically utilized by propionate- and butyrate-producing species and therefore do not accumulate at high levels in the colon.^205^ ^,^ ^206^ Depletion of these species and their metabolic by-products leads to increased intestinal permeability, facilitating the translocation of bacteria and LPS into the systemic circulation.^207^ Hematogenous dissemination of these PAMPs to the genital tract, or the resulting systemic inflammatory response, can promote genital tract inflammation.^208^ ^,^ ^209^ Similarly, bile acid derivatives and tryptophan metabolites produced in the gut enter the systemic circulation and have been found to alter cervical epithelial differentiation and immune surveillance.^65^ ^,^ ^210-213^ These molecular connections highlight how diet-microbiota interactions in the gut propagate their effects onto distal mucosal surfaces.

Taken together, these studies support selected rectal-vaginal co-carriage, extravaginal reservoir models, indirect metabolite-mediated pathways, and adjacent gut-urinary connections, while also showing that cross-site concordance is context-dependent and not necessarily directional (Table 2). Accordingly, the proposed gut–genitourinary axis should be interpreted as a biologically plausible framework requiring paired longitudinal and strain-resolved validation rather than as evidence of uniform microbiome-wide transfer.

**Table 2.** Structured synthesis of evidence supporting selected gut–genitourinary links and counter-evidence limiting broad cross-compartment inference.

Panel A. Evidence supporting selected components of the proposed axis					
Study	Population/model	Design and sampled sites	Principal finding	Interpretation	Ref.
Antonio et al., 2005	Women attending clinic	Cross-sectional human cohort; rectal and vaginal colonization assessed.	Co-colonization of vagina and rectum with H2O2-producing Lactobacillus species was associated with the lowest BV prevalence.	Supports a rectal reservoir or protective co-colonization model for selected taxa; does not establish directionality.	[214]
El Aila et al., 2009a; El Aila et al., 2009b	Pregnant women; 35-37 weeks for GBS substudy	Cross-sectional paired vaginal and rectal sampling with genotyping.	Shared species and identical genotypes were recovered from both sites in many dual-site carriers.	Supports cross-site carriage of selected strains; simultaneous sampling cannot determine source or direction.	[185,189]
Marrazzo et al., 2012	Women initially without BV followed for incident BV	Prospective cohort with oral, anal, and vaginal sampling.	Baseline oral or anal carriage of selected BV-associated bacteria was associated with subsequent BV acquisition.	Supports pre-existing extravaginal reservoirs preceding BV; transmission was not strain-resolved.	[190]
Fredricks et al., 2022	Women who have sex with men	Prospective cohort assessing vaginal and extra-vaginal bacterial colonization.	Extra-vaginal colonization patterns were associated with incident BV risk.	Strengthens prospective reservoir evidence in a second population; route and direction remain unresolved.	[215]
Bommana et al., 2025	Women with genital Chlamydia trachomatis treated with azithromycin	Paired longitudinal shotgun metagenomics of vaginal, endocervical, and rectal samples before and after treatment.	Azithromycin produced site-specific taxonomic, functional, and resistome changes across sampled sites.	Supports coordinated multi-site antimicrobial effects; does not prove gut-first causality.	[192]
Liu et al., 2025	Pregnant participants from ECHO cohorts	Multi-cohort paired maternal vaginal and fecal microbiota analysis.	Maternal vaginal and fecal microbiota in later pregnancy both contributed to infant fecal microbiota development.	Supports paired pregnancy cross-compartment relevance; 16S-level data do not establish strain-resolved maternal gut-vaginal transfer	[216]
Worby et al., 2022	Women with and without recurrent UTI	Year-long longitudinal multi-omics with gut, urinary, and host immune profiling.	Recurrent UTI was linked to gut dysbiosis, systemic immunity, and frequent gut-to-bladder strain transmission.	Supports a broader gut-genitourinary framework, but it is urinary rather than direct gut-vaginal evidence.	[217]
Horseman et al., 2026	Postmenopausal women with recurrent UTIs	Cross-sectional comparison of rectal, vaginal, and historical urine organisms and antimicrobial-resistance profiles.	Organism and antimicrobial-resistance concordance was observed across rectal, vaginal, and urinary reservoirs.	Supports adjacent rectal-vaginal-urinary reservoir communication; lacks whole-genome strain confirmation.	[194]
Li et al., 2025	Genetic and biobank-based vaginitis analysis	Mendelian randomization with plasma-metabolite mediation analysis.	Gut taxa were associated with vaginitis risk or protection, with candidate plasma-metabolite mediators.	Supports an indirect metabolite-mediated gut-vaginal pathway; does not test paired microbiome concordance.	[200]
Perez et al., 2025	Healthy women receiving oral Lactobacillus gasseri CECT 30648	Randomized probiotic intervention with strain-specific qPCR.	The orally administered strain was detected in vaginal samples in a subset of treated participants.	Shows oral administration can be followed by vaginal recovery of a strain; anatomical route and spontaneous transfer frequency remain unknown.	[218]
Karpinets et al., 2020	Mouse model with oral antibiotics	Mechanistic animal study with gut and vaginal microbiome profiling.	Antibiotics remodeled gut and vaginal microbiomes and increased sharing of gut taxa with vaginal communities.	Supports experimentally induced multi-site remodeling; human generalizability is limited.	[219]

Panel B. Discordant evidence and constraints on broad cross-compartment inference					
Study	Population/model	Design and sampled sites	Principal finding	Interpretation	Ref.
Bommana et al., 2022	Fijian women with and without Chlamydia trachomatis	Pilot paired shotgun metagenomics of endocervical, vaginal, and rectal samples.	Rectal, vaginal, and endocervical communities were largely site-specific, with selected cross-site similarities.	Substantial site specificity argues against uniform cross-site concordance; selected similarities may reflect sharing in some participants.	[191]
Goltsman et al., 2018	Pregnant women	Longitudinal strain-resolved metagenomics across gut, vaginal, and oral sites.	The same species could occur across gut and vagina while the corresponding strains were genetically divergent.	Species-level overlap does not establish shared strains or direct transmission.	[195]
Song et al., 2025	Late-pregnancy women with or without Group B Streptococcus colonization	Paired vaginal and stool sampling with genus-level correlation analysis.	Gut-vaginal correlations were weak, and none remained significant after correction for multiple testing.	Cross-site taxonomic concordance is not consistently reproducible and appears context-dependent.	[220]
Bar et al., 2023	Women with inflammatory bowel disease and healthy controls	Cross-sectional vaginal microbiota comparison stratified by IBD diagnosis, disease activity, and menopausal status.	Vaginal community composition did not differ significantly by IBD diagnosis or activity; menopausal status had a stronger association.	A major gut inflammatory disease does not necessarily produce a predictable vaginal signature; local and life-stage factors may dominate.	[221]

### 2.2. Distinct microbiomes under shared endocrine control

The gut microbiome is increasingly understood in a system-wide framework that includes the gut-brain axis and potential neuroendocrine links to the cervicovaginal microbiota.^222^ For example, vagal afferent signaling, hypothalamic-pituitary pathways, and circulating microbially derived metabolites can each influence reproductive physiology.^223^ Stress activation of the hypothalamic-pituitary-adrenal axis increases intestinal permeability and remodels the gut microbiome, while also being associated with disrupted menstrual cyclicity and potentially weakened cervicovaginal immunity.^224–226^ Together, these effects lend support for the idea of a complex microbial and neuroendocrine network that integrates digestive and genitourinary function.

The gut microbiome also interfaces with non-gonadal endocrine systems, including the thyroid axis, through immune regulation, intestinal permeability, micronutrient availability, and microbial effects on thyroid hormone metabolism.^167^ In the gut, endocrine hormones influence microbial composition by shaping nutrient availability, motility, and host immunity. Gastrointestinal hormones such as GLP-1, ghrelin, and peptide YY regulate gastric acid secretion, gut motility, and intestinal transit time, indirectly structuring microbial niches.^227-229^ Sex steroids exert even stronger effects: estrogens and androgens alter hepatic bile acid pools, which in turn shift microbial balance.^230^ ^,^ ^231^ For example, estrogen supplementation enriches butyrate-producing *Firmicutes*, whereas androgen dominance favors *Bacteroides* and *Prevotella*, patterns observed in hyperandrogenic states such as PCOS.^232-234^

The vaginal microbiome is more directly regulated by sex steroids. Estrogen drives glycogen accumulation in the epithelium, promoting *Lactobacillus* fermentation to lactic acid and sustaining a low-pH environment protective against opportunists.^235-237^ Declines in estrogen, as during menopause, reduce *Lactobacillus* abundance and favor BV–associated taxa such as *Gardnerella* ^238^. Progesterone has been associated with a stabilizing effect, with luteal-phase levels linked to reduced diversity and maintenance of *Lactobacillus* dominance.^239^ Across the menstrual cycle, longitudinal studies show microbial diversity tends to increase during menses and stabilize during the follicular and luteal phases.^240^ Despite observed luteal-phase stability, direct progesterone effects are less consistent than those of estradiol.^93^ ^,^ ^112^ ^,^ ^241^

Across human cohorts, higher estrogen status is frequently associated with greater gut microbial diversity, whereas in the vagina, it is associated with *Lactobacillus*-dominant, low-diversity communities.^161^ ^,^ ^238^ ^,^ ^242-244^ This inverse pattern suggests coordinated endocrine effects across niches, although a strict linear relationship between niches has not been demonstrated. Observations during clinical interventions also support a dual endocrine influence on mucosal microbiota. In the vagina, topical estrogen and several hormonal contraceptives are associated with higher *Lactobacillus* abundance and greater community stability, although effects vary by method.^112^ ^,^ ^245^ ^,^ ^246^ Some evidence suggests hormonal contraception may also alter gut beta diversity; vaginal estriol increases *Lactobacillus* and *Bifidobacterium* and lowers pH in postmenopausal women, and in PCOS elevated gut *β*-glucuronidase has been linked to sex-hormone imbalance.^247-250^ In the gut, exogenous sex steroids and shifts in steroid metabolism during endogenous imbalances can alter community composition and function, but the magnitude and direction are context-specific.^251-253^ Taken together, these findings support a model in which the gut and vaginal microbiota are independently structured yet jointly responsive to shared hormonal cues.

### 2.3. Correlation between dysbiosis in disease states

Across many conditions, dysbiosis is observed in both the gut and cervicovaginal niches. These co-occurring shifts do not, by themselves, establish cross-site causation. More plausibly, shared disease processes perturb both ecosystems in parallel through systemic inflammation, endocrine disturbance, immune remodeling, epithelial barrier dysfunction, medication exposures (including antibiotics and hormones), and altered metabolite pools such as bile acids and tryptophan derivatives.^254-256^ Framing the correlation this way clarifies that common host drivers can yield coordinated microbial change without requiring one site to directly control the other.

PCOS illustrates a systemic driver producing parallel but compartment-specific effects; hyperandrogenism and insulin resistance create an inflammatory milieu that destabilizes both intestinal and cervicovaginal communities.^257^ Gut microbial alterations are repeatedly reported in PCOS, including differences in diversity and enrichment of taxa linked to metabolic inflammation and insulin resistance.^258-262^ The mechanism by which microbial *β*-glucuronidase activity affects women with PCOS involves its action in deconjugating steroid hormones in the intestine.^263^ This process enhances enterohepatic recycling of estrogens and androgens, amplifying systemic hormone imbalance that feeds back on both host metabolism and mucosal ecosystems.^249^ ^,^ ^264^ In connection with these hormone changes, clinical evidence suggests that women with PCOS more frequently display vaginal community shifts away from *Lactobacillus* dominance.^265^ ^,^ ^266^ These patterns are evidence that systemic drivers in pathologies like PCOS—such as hyperandrogenism, insulin resistance, and low-grade inflammation—are upstream determinants of “parallel” dysbiosis in the gut and vagina; this axis is not a simple one-way causal chain.^258^ ^,^ ^260^ ^,^ ^267-269^ Experimental microbiota-transfer studies further strengthen the gut-reproductive component of this model: transfer of dysbiotic gut microbiota from women with PCOS into antibiotic-treated mice induced reproductive and metabolic abnormalities, while related mechanistic work implicates gut microbiota-dependent bile acid and IL-22 signaling in PCOS-like endocrine disruption.^270^ ^,^ ^271^ Microbiome-targeted interventions have also been studied in PCOS, where synbiotic or probiotic formulations have been associated with improvements in insulin sensitivity, androgen-related measures, lipid profiles, or oxidative-stress markers in selected clinical studies, although these trials generally do not establish paired gut-vaginal microbial mechanisms.^272-276^

Similar patterns of parallel dysbiosis are observed across the gut-vaginal axis in endometriosis.^277^ Case-control and cohort studies report altered gut microbial composition in affected women, with significant shifts in taxa also implicated in immune activation and estrogen metabolism.^278-280^ Balla et al. reported that women with endometriosis or subfertility exhibit lower *Lactobacillus* dominance and greater representation of inflammatory anaerobes in the cervicovaginal microbiota.^281^ ^,^ ^282^ These findings can be explained by systemic processes characteristic of endometriosis: chronic inflammation, ectopic estrogen signaling, and immune dysfunction, which together reshape both intestinal and cervicovaginal mucosa.^283-285^

Metabolic diseases such as obesity and type 2 diabetes are associated with gut dysbiosis, increased intestinal permeability, and inflammatory metabolic signaling.^286-289^ Observational studies associate higher body mass index and hyperglycemia with greater odds of bacterial vaginosis or *Lactobacillus* depletion, plausibly via altered mucosal immunity, glycogen handling in the vaginal epithelium, and systemic cytokine profiles.^290-294^ These host states can concurrently destabilize gut and vaginal ecosystems.^286^ ^,^ ^294^

Gut dysbiosis is a feature of IBD; however, comparative analyses of women with IBD versus controls show that vaginal CSTs are often more strongly shaped by menopausal status than by IBD diagnosis or activity.^295^ This implies that not every systemic inflammatory disease produces predictable genital tract dysbiosis.^221^ At the same time, studies in pregnancy suggest that women with IBD can harbor cervicovaginal taxa linked with adverse outcomes more frequently than healthy comparators.^296^ ^,^ ^297^ Systemic inflammation and clinical exposures may influence risk, but the magnitude and direction of vaginal changes may need to account for life stage and local mucosal factors.^221^ ^,^ ^296^

HIV infection is associated with persistent gut dysbiosis and barrier defects, even with effective therapy.^298^ ^,^ ^299^ Women with HIV show shifts in cervicovaginal diversity, resulting in diminished *Lactobacillus* populations; this further correlated with inflammation and adverse gynecologic outcomes.^300^ ^,^ ^301^ The coincidence of intestinal and cervicovaginal dysbiosis in HIV may reflect systemic immune dysregulation and antiretroviral exposures as common pressures on both microbial communities.^299^ ^,^ ^301^

Apart from pathology, medication exposures can simultaneously disrupt vaginal and gut microbiota, pointing to shared regulatory influences.^302^ ^,^ ^303^ Non-antibiotic medications may also confound microbiome associations; for example, proton pump inhibitor exposure has been associated with altered gut microbial composition, underscoring the need to account for medication history when interpreting cross-compartment dysbiosis patterns.^304^ Broad-spectrum antibiotics rapidly deplete gut commensals and can produce durable alterations in diversity and function.^305^ Clinically, antibiotic use often precedes vulvovaginal candidiasis, and post-treatment recurrence of BV reflects biofilm tolerance and antimicrobial resistance.^306-308^ These observations are consistent with multiple routes of coupling between niches: direct, potentially bidirectional microbial exchange across adjacent mucosae and indirect systemic pathways involving immune, endocrine, barrier, and pharmacologic effects.^190^ ^,^ ^309^

A plausible mechanism for the co-occurrence of gut and vaginal dysbiosis in disease states is systemic immune activation coupled with shifts in metabolic mediators (e.g., SCFAs, bile acids, and tryptophan derivatives).^310^ ^,^ ^311^ Chronic inflammatory conditions such as PCOS, endometriosis, and HIV are characterized by persistent low-grade inflammation and dysregulated cytokine signaling.^312-314^ This systemic immune tone can weaken epithelial barriers in both the intestine and the cervicovaginal tract, promoting microbial translocation, reducing *Lactobacillus* stability, and permitting overgrowth of opportunistic taxa.^180^ ^,^ ^315^ ^,^ ^316^

Additionally, systemic inflammation redirects metabolic resources.^317^ Increased glucose utilization by immune cells and altered glycogen storage in mucosal tissues reduce substrates available for *Lactobacillus* in the vagina, while inflammatory damage to intestinal epithelia alters nutrient absorption and reshapes the gut microbiome.^318-320^ Parallel dysbiosis thus reflects a host-mediated redistribution of resources under inflammatory stress.^315^ ^,^ ^320^ Together, these findings suggest that immune–endocrine–metabolic feedback loops might explain why disease states often produce correlated microbial disturbances across gut and vaginal ecosystems.

Evidence across studies is not uniformly concordant. Some paired rectal and vaginal studies identify overlapping taxa or co-colonization, whereas metagenomic comparisons have also reported substantially site-specific microbial populations and functions.^185^ ^,^ ^191^ Similarly, co-occurring dysbiosis in conditions such as PCOS, inflammatory bowel disease, pregnancy, and chronic infection may reflect shared hormonal, immune, metabolic, or treatment-related pressures rather than direct microbial transfer between sites.^35^ ^,^ ^258^ ^,^ ^321^ These findings do not negate the proposed cervicovaginal-gut axis; rather, they indicate that its strength and mechanism are likely context-dependent.^191^ ^,^ ^195^ Taxonomic overlap should therefore not be interpreted as evidence of directionality, and parallel dysbiosis should not be assumed to imply that one microbial compartment can reliably predict another.^322^

A related but distinct evidence stream comes from the HPV and cervical cancer literature, where support is stronger for local cervicovaginal microbe-host effects than for cross-compartment gut-vaginal inference.^36^ ^,^ ^138^ These studies are therefore interpreted here as evidence for local disease mechanisms and mucosal immune modulation, rather than as direct support for gut-vaginal transfer, cross-site directionality, or systemic surrogate validity.

## 3. Vaginal microbiota during pregnancy

We dedicate a full section to pregnancy because it represents a unique physiologic state in which hormonal, immune, and metabolic changes occur concurrently and exert broad effects across multiple microbial niches. Gestation is therefore a useful model for examining how host-mediated processes shape the gut, vaginal, and systemic microbiomes in parallel, even though most available evidence derives from compartment-specific studies rather than paired longitudinal sampling. By situating pregnancy-associated dysbiosis within this shared physiologic context, this section highlights clinically relevant associations with outcomes such as preterm birth, preeclampsia, and gestational diabetes, while avoiding assumptions of direct microbial exchange between body sites.

### 3.1. Pregnancy-associated microbiome shifts across body sites

Pregnancy involves a wide range of physiological transformations that enable the maternal body to support fetal development. These transformations are primarily driven by endocrine, metabolic, neuroendocrine, and immune adaptations that influence systemic processes ranging from digestion to psychological function and behavior, with particularly pronounced effects on mucosal surfaces and the genital tract environment.^323^ While physiologic changes during pregnancy substantially alter the composition and function of the vaginal microbiome, the vaginal microbiota may also exert reciprocal effects on immune function, hormonal balance, and the overall reproductive environment.^324^ As a result, the vaginal microbiome becomes embedded within a broader interaction network that distinguishes pregnancy from the nonpregnant state.

Compared with nonpregnant women, pregnancy is marked by lower vaginal microbial diversity, greater compositional stability, and enrichment of *Lactobacillus* species.^325^ In a retrospective longitudinal case-control study, Romero et al. reported more stable communities in pregnant women with higher relative abundance of *Lactobacillus vaginalis*, *L. crispatus*, *L. gasseri*, and *L. jensenii*, alongside reduced prevalence of 22 other phylotypes.^326^ These findings were extended by Marangoni et al., who sampled healthy pregnant Caucasian women across the first, second, and third trimesters and observed progressive declines in diversity with increasing dominance of lactobacilli.^327^ ^,^ ^328^ Across multiple cohorts, convergent evidence indicates that gestation favors *Lactobacillus* dominance, greater temporal stability, and reduced richness.^329^

Vaginal communities are commonly grouped into CSTs by their dominant taxa: CST I (*Lactobacillus crispatus*), CST II (*L. gasseri*), CST III (*L. iners*), and CST V (*L. jensenii*). These *Lactobacillus*-dominant states are generally considered protective.^8^ In a European pregnancy cohort, CST I was most frequent (40%), followed by CST III (27%), CST V (13%), and CST II (9%); CST IV was least common overall (8%) and rare during pregnancy (2%).^330^ Across studies, CST I and CST III consistently predominate in pregnancy, with modest variation in proportions.^331^ ^,^ ^332^ Longitudinal studies also show a shift from CST IV toward *Lactobacillus*-dominant states from early gestation through the third trimester, along with transitions among Lactobacillus-dominant CSTs.^333-335^

These pregnancy-associated vaginal microbiome patterns emerge within a tightly regulated neurohormonal and immune context. The hypothalamic-pituitary-adrenal (HPA) axis regulates systemic hormone production and stress responses,^336^ and during pregnancy placental corticotropin-releasing hormone levels rise progressively, stimulating maternal ACTH secretion and increasing cortisol production.^337^ Estrogen also increases during pregnancy to roughly fifty to one-hundred times the levels observed across a normal menstrual cycle.^338^ ^,^ ^339^ Elevated estrogen drives proliferation and differentiation of the vaginal epithelium and expands epithelial glycogen stores,^340^ which are cleaved by *α*-amylase into fermentable substrates that *Lactobacillus* species convert to lactic acid; thereby lowering vaginal pH, favoring *Lactobacillus* dominance, and reducing overall diversity.^341-343^ Together, high estrogen levels and a lactic-acid-rich environment strengthen the mucosal barrier, support vaginal health, and are associated with reduced risk of STIs and certain cancers.^344^

The HPA axis governs stress response and remains highly active during pregnancy.^345^ In the second and third trimesters, maternal cortisol rises to roughly two to three times that of nonpregnant levels and typically returns to baseline within days postpartum.^346^ Stress signaling intersects with the microbiota, illuminating neuroendocrine pathways that link brain activity with gut and vaginal ecosystems.^347^ ^,^ ^348^ In an animal model, Wrenn et al. showed that co-administration of cortisol or deoxycorticosterone with 17β-estradiol attenuated the estrogen-driven vaginal glycogen response, suggesting that stress steroids can counter local estrogenic effects on the mucosa.^349^ Human and animal studies further associate maternal stress with shifts in gut and vaginal communities and with inflammatory and mental health phenotypes.^350-353^

Emerging data link affective symptoms in pregnancy to shifts in the vaginal microbiome, consistent with neurochemical and hormonal modulation of this niche. In a third-trimester cohort of 275 participants, Scheible et al. ^354^ found that higher depressive symptoms were associated with specific taxa and with a community profile lacking single-species dominance; this profile was enriched for pathways producing serotonin and dopamine precursors from L-tryptophan and L-phenylalanine. In a separate study of 140 women, Nel et al. ^355^ reported that higher Edinburgh Postnatal Depression Scale scores correlated with *Lactobacillus iners* dominant communities and lower abundance of the more classically protective *Lactobacillus crispatus*. Collectively, these findings support a bidirectional relationship between neurohormonal signaling, mood, and the vaginal microbiome during pregnancy.^356^

Pregnancy also reshapes immunity in the cervicovaginal tract, and the vaginal microbiota both responds to and helps drive these changes.^357^ Around implantation, local immune responses are tempered to permit embryo attachment, and some studies note a transient shift away from strict *Lactobacillus* dominance toward a more heterogeneous community resembling nonoptimal states.^358^ ^,^ ^359^ As gestation progresses, immune tone and rising estrogens favor a return to *Lactobacillus*-dominant, lower-diversity communities, a pattern consistent with the broader pregnancy phenotype and likely reinforced by hormonal cues.^360^

During pregnancy, *Lactobacillus* ferments glycogen-derived sugars to lactic acid, which lowers vaginal pH and suppresses opportunists (*Enterobacteriaceae, Streptococcus, Staphylococcus, Escherichia coli*).^89^ ^,^ ^361^ Lactic acid contributes antimicrobial activity and modulates immunity by limiting monocyte and CD8 T cell differentiation, inhibiting dendritic cell maturation, and promoting M2 macrophage polarization.^362^ When lactic acid is low, inflammatory mediators increase, including IL-6, IL-8, IL-1α, IL-1β, and MIP-1α or MIP-1β, and communities are less *Lactobacillus*-dominant.^360^ ^,^ ^363^ ^,^ ^364^

Distinct microbial communities contribute to the metabolome in ways that may influence the maternal immune system. In a cohort of 53 pregnant women, Oliver et al. grouped cervicovaginal communities as *Lactobacillus crispatus* dominant, *L. iners* dominant, or diverse non-*Lactobacillus* states and found that indole-3-lactate (ILA), a tryptophan-derived metabolite, was enriched in *L. crispatus* dominant profiles.^365^ *L. crispatus* appears to produce ILA, which has antimicrobial activity and also ligates the aryl hydrocarbon receptor, a host regulator of epithelial integrity and mucosal immune responses, providing a metabolite-to-immunity link specific to pregnancy communities.^366-368^

Although pregnancy-related changes in both vaginal and gut microbiomes are well documented, most studies examine these compartments independently, and few include paired longitudinal sampling across body sites.^369^ Consequently, similarities observed across microbial niches during pregnancy are more plausibly attributed to shared host-mediated hormonal, immune, and metabolic drivers, rather than evidence of direct inter-site microbial exchange. Pregnancy, therefore, represents a state of parallel, host-driven microbiome remodeling across body sites, while highlighting the need for integrative study designs to resolve potential inter-compartment interactions.

### 3.2. Dysbiosis in disease states and adverse pregnancy outcomes

Vaginal dysbiosis, particularly CST IV and its subtypes, increases risk for genital infections, including BV and aerobic vaginitis, and is associated with higher susceptibility to STIs.^370-374^ Dysbiosis is also associated with adverse obstetric outcomes such as miscarriage and preterm birth.^375-377^

GDM shows consistent signals of altered vaginal ecology, although taxa-level findings vary across cohorts.^378^ Studies report enrichment of non-*Lactobacillus* genera and higher rates of vaginal infection, especially vulvovaginal candidiasis, in women with GDM, along with reduced prevalence of protective *Lactobacillus* flora.^379-383^ Taken together, these data support the potential for early-pregnancy microbiome profiles to contribute to GDM risk stratification.^384^

PE and related hypertensive disorders of pregnancy are also linked to altered vaginal communities.^385^ Reports include higher community diversity with depletion of *Lactobacillus* and enrichment of anaerobes such as *Gardnerella, Prevotella, Fannyhessea, Dialister*, and *Sneathia*, as well as specific findings such as increased *Prevotella bivia* in severe preeclampsia (SPE).^386^ ^,^ ^387^ In a nested case–control study, pregestational communities dominated by *Lactobacillus crispatus* were associated with lower subsequent risk of hypertensive disorders.^388^

Across conditions, dysbiosis in pregnancy aligns with systemic inflammatory tone, endocrine disruption, and barrier dysfunction that are central to the cervicovaginal-gut axis, although most available data remain associative and do not establish directionality or direct inter-site microbial exchange.^389^ These patterns position the vaginal microbiome as both a potentially modifiable target and a candidate biomarker platform for early identification of at-risk pregnancies and for testing multi-modal interventions, including diet, probiotics, microbiota-based therapies, and hormone management.^390^

### 3.3. Systemic microbiome variation during pregnancy and links to the gut–vaginal axis

Pregnancy reshapes multiple microbial ecosystems.^391^ In the gut, community structure shifts from the first to the third trimester concurrent with hormonal changes, such as rising progesterone levels.^392-394^ In early pregnancy, gut profiles showing lower Bifidobacterium and Streptococcus have been associated with subsequent preterm birth, while reduced SCFA-producing taxa have been linked to PE and GDM.^395-400^ By the third trimester, studies report higher *Bifidobacterium, Pseudomonadota, and Actinomycota*, lower overall diversity, and more opportunists, with links to preterm birth, PE, and GDM.^401^

GDM shows consistent alterations in gut composition, although specific taxa vary by cohort.^402^ Reported patterns include increased *Firmicutes* with decreased *Bacteroidetes* and *Actinobacteria* from the second to the third trimester, as well as enrichment of *Collinsella, Rothia*, and *Desulfovibrio; Collinsella* correlates with higher fasting insulin and insulin resistance.^403^ ^,^ ^404^ After delivery, women with prior GDM retain distinctive gut signatures for months, including enrichment of *Bavariicoccus, Clostridium* sensu stricto, *Bacteroides, Veillonella, and Prevotella* with relative depletion of *Firmicutes*.^405^ Metagenomic work further identifies *Bacteroides* and *Klebsiella variicola* enrichment in GDM and highlights linkage groups that correlate with blood glucose, suggesting utility for risk stratification.^406^

Together, these gut findings align with the cervicovaginal patterns described during pregnancy and support a systems-based view. However, longitudinal human studies that concurrently profile gut and cervicovaginal microbiomes during pregnancy are scarce, limiting the ability to assess coordinated inter-site microbial dynamics.^35^ ^,^ ^407^ Hormonal signaling and immune tone change across gestation and may couple gut signals to the cervicovaginal niche through metabolites such as SCFAs, bile acid derivatives, and tryptophan products, as well as through estrogen recycling.^29^ ^,^ ^408^ This coherence across compartments is consistent with a systems-based framework for gut and cervicovaginal interactions during pregnancy and sets the stage for the clinical questions considered next.

## 4. Clinical significance

Having outlined the biological mechanisms linking the vaginal and gut microbiomes, two central questions arise: how close current approaches are to producing reliable, actionable forecasts, and whether vaginal profiles can serve as practical surrogates for more complex systemic readouts. Addressing these questions requires evaluating both the capabilities and limitations of current predictive models, as well as the feasibility of scaling sampling strategies beyond research settings.

### 4.1. A computational roadmap for cross-compartment microbiome prediction

Currently, microbiome science is transitioning from descriptive cataloging to predictive modeling. Research has moved beyond simply cataloging “who is there,” yet remains far from enabling diagnostic-level predictions from a single swab or droplet of blood. The field has entered a modeling era, characterized by digital twins of gut communities,^409^ organ-resolved human reconstructions, and machine learning models trained on metagenomes (see boxed definitions below). These tools are powerful in research contexts but remain premature for routine clinical application. A central question is what it would take to progress from the current stage to a future in which a small local metabolite shift, such as in vaginal fluid, saliva, or a finger-prick of blood, could reliably forecast outcomes as significant as neurologic decline or cancer risk.^410^

**Digital twins**: Computational replicas of biological systems that integrate multi-omic data and mechanistic models to simulate and predict how an individual’s microbiome, organs, or whole body will respond to diets, drugs, or disease.

**Organ-resolved whole-body human metabolic reconstruction:** A computational model of human metabolism that integrates all known metabolic reactions (enzymes, transporters, metabolites) with organ-specific resolution, partitioning the network into compartments representing multiple organs and tissues (e.g., liver, muscle, brain, gut, kidney, adipose, blood). It incorporates inter-organ connections through metabolite fluxes in blood, lymph, and other shared pools, enabling exchange of nutrients and signaling molecules. Together, these features create a virtual human metabolic network capable of simulating how diet, disease, or drugs impact metabolism across the entire body.

**Atom-mapped**: A description of a biochemical reaction in which each atom in the reactants is explicitly tracked to its corresponding position in the products. Atom mapping shows how individual atoms (such as carbon, hydrogen, oxygen, or nitrogen) are rearranged, conserved, or lost during the reaction, enabling precise tracing of molecular transformations, isotope labeling studies, and accurate construction of metabolic network models.

**Spatially explicit agent-based metabolism:** Tracks cells, local metabolites, and space; each time step solves FBA per agent, so you get emergent cross-feeding, mucus layer gradients, and biofilm-like structure. A published BacArena gut demo used a seven species consortium and mucus glycan gradients to replicate known niche formation.

The most advanced models currently available are constraint-based digital twins capable of simulating an individual’s gut community.^411^ ^,^ ^412^ For instance, AGORA2 provides strain-level metabolic reconstructions for more than 7,000 microbes, curated down to the atom-mapped reaction level.^411^ MICOM incorporates these reconstructions with dietary inputs, abundance profiles, and optimization routines to predict metabolite fluxes.^413^ A number of new platforms, pipelines, and analysis tools are explored in Table 3. These models can already reproduce some clinically observed features, such as reduced butyrate in type 2 diabetes and its partial restoration with metformin, as well as interindividual differences in how gut microbes metabolize chemotherapy drugs.^414^ ^,^ ^415^ At the whole-body scale, “organ-resolved” reconstructions, like Harvetta, connect dozens of tissues through blood and lymph, enabling simulation of how microbial metabolites enter circulation and impact organs beyond the gut.^416^

**Table 3.** Computational and experimental platforms for implementing cross-compartment microbiome prediction.

Platform(s)	Role in workflow	Core capability	Application to proposed gut–cervicovaginal program	Ref.
AGORA2	Strain and reaction reconstruction	Genome-scale metabolic reconstructions for 7,302 human-associated microorganisms, curated to the atom-mapped reaction level.	Defines organism-specific metabolic capacities that can be linked to species detected across gut, vaginal, cervical, or rectal samples.	[411]
MICOM	Community metabolic modeling	Combines abundance profiles, diet, and microbial metabolic reconstructions to estimate community-level metabolic fluxes.	Predicts SCFA production, drug-microbe effects, metabolic consequences of candidate probiotic addition, and other community-level outputs relevant to systemic signaling.	[413]
MIMOSA2	Microbiome–metabolome integration	Integrates paired microbiome and metabolomics data with metabolic reference networks to identify metabolites, taxa, and reactions linked to observed variation.	Tests whether SCFAs, bile acids, tryptophan products, or hormone-related metabolites are plausibly microbiome-governed mediators across compartments.	[417]
microbeMASST	Microbial metabolite annotation and producer attribution	Searches known or unknown MS/MS spectra against a curated database derived from more than 60,000 microbial monocultures and links spectra to likely microbial producers.	Helps assign metabolites detected in stool, plasma, vaginal fluid, or experimental systems to candidate producing taxa and validate model-predicted metabolic nodes.	[418]
COMETS; BacArena	Spatial and temporal microbial ecology	Simulates microbial growth, nutrient gradients, cross-feeding, competition, and spatial organization over time.	Models local niche structure such as gut mucus gradients, vaginal biofilm-like organization, and spatially constrained metabolite exchange.	[45]
MDSINE2	Ecological interaction and perturbation inference	Bayesian framework that learns compact, interpretable ecosystem-scale dynamical models, microbial interaction modules, and responses to perturbations from time-series data.	Could infer whether antibiotics, hormonal shifts, diet, or pregnancy transitions alter specific interaction networks within and across longitudinal gut and cervicovaginal datasets.	[419]
MicroProphet	Temporal community forecasting	Machine-learning framework for predicting personalized microbiome trajectories from incomplete longitudinal data.	Could test whether vaginal CST transitions, gut shifts, or paired cross-compartment dynamics can be forecast longitudinally.	[420]
Harvetta/whole-body metabolic models	Host–microbe systems integration	Organ-resolved whole-body metabolic reconstruction linking microbial metabolism with host physiology, blood, lymph, and tissue compartments.	Provides a host whole-body metabolic framework for tracing gut-derived microbial metabolites through systemic circulation and could be extended to incorporate cervicovaginal microbial inputs.	[416]
DEBIAS-M	Processing-bias correction and harmonization	Learns microbe- and batch-specific correction factors to improve cross-study microbiome prediction and domain adaptation.	Supports multicohort cervicovaginal–gut modeling by reducing technical artifacts from processing, sequencing, and study-specific batch effects.	[421]
MetaPhlAn 4/StrainPhlAn 4	Species- and strain-level metagenomic profiling	Profiles species-level genome bins from shotgun metagenomes and reconstructs highly resolved sub-species phylogenies, including uncharacterized taxa.	Standardizes profiling across paired gut and cervicovaginal samples and identifies candidate strains for cross-site comparison or transmission analysis.	[422,423]
ChronoStrain	Longitudinal strain tracking	Time-aware Bayesian strain-profiling approach for estimating strain presence and abundance across longitudinal metagenomic samples.	Helps distinguish candidate cross-site strain sharing from species-level taxonomic overlap in paired gut, rectal, vaginal, cervical, or longitudinal time series.	[424]
PacBio HiFi/long-read metagenomics; metaMDBG	Genome-resolved measurement and assembly	Assembles long accurate metagenomic reads into more complete genomes and improves recovery of strains, viruses, plasmids, and difficult genomic regions.	Tests cross-site strain identity, mobile-element sharing, and functional variation beyond marker-gene or species-level overlap.	[425]
GenomeOcean	Genome foundation modeling	Large genome foundation model trained on metagenomic assemblies to learn DNA sequence patterns directly from raw sequence space.	May identify unannotated genes, hidden regulatory patterns, or functional sequence features missed by curated metabolic reconstructions.	[426]
BiomeGPT	Species-level microbiome foundation modeling	Transformer-based foundation model pretrained on more than 13,300 human gut metagenomes spanning 32 phenotypes to learn species-level microbiome representations.	Could provide reusable gut microbiome embeddings for biomarker discovery, disease stratification, and hypothesis generation in cross-compartment prediction.	[427]
Gut-on-chip/cervical microphysiological systems	Experimental causal validation	Microfluidic tissue models combine human epithelium, flow, microbiota, and selected host or pathogen components; recent systems incorporate patient fecal microbiota or cervical microbiota and genital pathogens.	Allows controlled testing of model-predicted host–microbiome responses, barrier effects, cross-compartment metabolites, and candidate interventions in physiologically relevant human systems.	[428,429]
Quantum-dot biosensors	Experimental model anchoring	Functionalized quantum-dot probes can detect selected metabolites, antibiotics, redox states, or cellular signals with spatial and temporal resolution.	Provides targeted validation of selected model-predicted analytes or cellular states in cells, organoids, or experimental systems; complements bulk metabolomics rather than replacing it	[430,431]

These models are computationally intensive, but not prohibitively so.^432^ Running MICOM on hundreds of strains across thousands of reactions requires solving linear programs with hundreds of thousands of variables.^433^ With solvers like Gurobi or CPLEX, these computations can be handled on a lab workstation or modest cluster in hours rather than weeks.^434-436^ Even complex spatial agent-based models can run on mid-range clusters, such as institutional computing clusters commonly available at universities and research laboratories.^45^ For this reason, most major institutions can already deploy these pipelines; they are no more computationally intensive than the analyses routinely performed in transcriptomics or imaging.^437^ What is missing is not raw computational capacity, but rather improved algorithms and, crucially, the appropriate types of data. Austin et al. developed DEBIAS-M, an interpretable processing-bias correction framework that learns microbe- and batch-specific correction factors and improves cross-study microbiome prediction, indicating that future cervicovaginal-gut models will require processing-aware harmonization rather than simple dataset pooling.^421^ In another study Austin, Pe’er, and Korem showed that leave-one-out and leave-*p*-out cross-validation can introduce distributional bias between training folds and held-out samples, supporting the need for participant-level and externally validated testing in small cervicovaginal microbiome prediction studies.^438^

Operating at the scale of large language models in artificial intelligence but trained on hundreds of gigabases of metagenomic DNA, foundation models like GenomeOcean contain billions of parameters and leverage hundreds of terabytes of microbial sequence data.^426^ ^,^ ^427^ ^,^ ^439^ These foundation models require multi-GPU clusters or ‘supercomputers’, because they are designed to capture patterns directly from raw sequence space. Mechanistic models like AGORA2 and MICOM have been applied to predict SCFA production, interindividual variation in drug metabolism, and probiotic engraftment, whereas foundation models are aiding in the annotation of unknown genes and the discovery of hidden regulatory patterns.^411^ ^,^ ^440^ ^,^ ^441^ These applications provide valuable mechanistic and functional insights, but they remain largely confined to research settings; they are not yet feasible for direct deployment in routine personal health prediction or clinical decision-making.^442^

Achieving clinical-grade predictive power will require three major advances. First, higher-resolution data are needed: absolute counts instead of relative abundances, multi-site sampling in the same individuals, and time series that capture dynamic shifts rather than static snapshots.^443-445^ Second, models must integrate metabolism, immunity, and endocrine signals rather than treating them separately; this is where coupling community flux balance analysis (FBA) with whole-body human reconstructions becomes essential.^446^ Third, interventions are required to test causality.^447^ Without perturbations such as microbiota transplants, targeted probiotics, or hormone modulation, only correlations can be observed.^112^ ^,^ ^219^ ^,^ ^448^ Causal inference requires experimental instruments, which are only now entering clinical trials.

A key benchmark is Alzheimer’s research.^449^ ^,^ ^450^ Several longitudinal gut microbiome studies have demonstrated unexpectedly high within-cohort accuracy for predicting progression from mild cognitive impairment to dementia; AUROC values ranging from 0.75–0.82 when microbiome data are combined with simple clinical features.^451-453^ However, these remain small, single-center studies, and external validation is lacking. A similar pattern of promising early signals requiring rigorous external validation is likely to apply to vaginal–gut inference. Although early cohorts reveal promising signals, generalizable prediction will require larger, multi-omic, multi-site datasets that are systematically collected and standardized.^454^ ^,^ ^455^

Metabolomics adds another layer to this trajectory, with quantum dots (QDs) beginning to show promise.^456^ ^,^ ^457^ Rather than relying solely on bulk assays, QDs can be engineered with enzymes, aptamers, or redox-sensitive probes to report local concentrations of metabolites such as glucose, lactate, or reactive oxygen species.^430^ ^,^ ^457^ Their narrow emission spectra enable multiplexing, allowing several metabolites to be tracked in parallel within the same sample, while their photostability supports long-term imaging in living systems.^430^ ^,^ ^431^ Although this approach does not yet provide full pathway reconstructions, it enables imaging of specific metabolic nodes *in situ*, even within organoids or single cells.^431^ Research groups are exploring QDs in STORM/PALM and multiphoton contexts,^458^ which could in theory generate nanometer-scale maps of metabolite dynamics at the organelle level.^459-461^ If paired with digital twins in the future, these targeted high-resolution readouts could eventually serve as experimental anchors, constraining simulations with live-cell metabolic data in ways that mass spectrometry alone cannot achieve.^462^

With currently available data and interventions, it may be possible to make directional inferences in tightly controlled cohorts over the coming years.^463^ Optimistically, cross-population validated predictions from subtle vaginal shifts to gut changes could become feasible within the next decade. Along this trajectory, the most immediate payoff is likely to come from identifying clinically useful proxies: cases in which the vaginal microbiome can serve as a readout of systemic tone when stool collection is impractical.^464^ ^,^ ^465^

Translationally, a cervicovaginal gut microbiota framework points to several potential therapeutic directions that aim to restore protective community structure, reduce inflammation, and modulate host drivers that shape both niches:

- Dietary modulation and targeted prebiotics to shift metabolite profiles and support barrier function.^11^ ^,^ ^23^ ^,^ ^27^ ^,^ ^466^ ^,^ ^467^
- Probiotics and live biotherapeutics, including strategies to restore *Lactobacillus*-dominant states.^18^ ^,^ ^147^ ^,^ ^468^ ^,^ ^469^
- Microbiota-based therapies, including emerging consortia-based approaches and microbiota transfer strategies.^448^ ^,^ ^470^
- Hormone-informed strategies that support estrogen-linked mucosal barrier integrity.^31^ ^,^ ^341^
- Adjunct antimicrobial or anti-inflammatory approaches paired with microbiome restoration to reduce recurrence and improve durability.^148^ ^,^ ^306^

### 4.2. Vaginal microbiome as a candidate proxy for systemic microbial states: future directions

The gut microbiome has emerged as a critical determinant of systemic health through its regulation of metabolism, immunity, and the gut–brain axis.^5^ However, stool-based microbiome profiling remains impractical for population-scale diagnostics. Although stool-based profiling is the current standard for gut microbiome analysis, multiple studies highlight the logistical and compliance challenges that limit its scalability for population-level application.^471^ ^,^ ^472^ Stool sample collection is cumbersome, results are highly sensitive to short-term dietary fluctuations and participant compliance is often poor.^473^ ^,^ ^474^ By contrast, the vaginal microbiome can be sampled rapidly, noninvasively, and reproducibly, raising the possibility that cervicovaginal profiles could serve as accessible proxies for gut states while also providing insight into systemic and neurologically relevant physiology.^475^

Feasibility and clinical utility are increasingly evident. Cervicovaginal sampling is already integrated into routine clinical workflows such as Pap smears, HPV screening, and BV testing**;** while self-collection kits enable reliable sampling outside of clinical settings.^201^ ^,^ ^476^ Microbial community profiling from these samples has demonstrated reproducibility across time and across laboratories, overcoming some of the barriers that limit gut microbiome studies.^93^ ^,^ ^477^ These practical features render the vaginal niche especially suitable for large-scale and longitudinal monitoring.

Operational accessibility should be distinguished from biological surrogacy.^478^ Existing studies demonstrate that vaginal samples can be collected and profiled reliably,^475^ but they do not yet establish that vaginal taxonomic or functional signatures consistently reproduce gut measurements or predict neurologic outcomes.^220^ Validation would require paired longitudinal gut and cervicovaginal sampling, adjustment for hormonal, behavioral, medication, life-stage confounders, strain- and metabolite-level concordance analyses, and external replication in independent populations.^192^ ^,^ ^479^ ^,^ ^480^ For clinical implementation, these validation studies will also require standardized vaginal microbiome-specific reference reagents, harmonized analytical protocols, and systematic metadata capture to improve reproducibility and comparability across cohorts.^481^ Until these criteria are met, vaginal profiles should be considered potential complementary biomarkers rather than replacements for stool-based profiling.

New technologies are accelerating translation into practice. qPCR-based assays targeting panels of vaginal bacteria achieve sensitivities and specificities above 95% for diagnosing BV, with same-day turnaround.^482^ Multiplex PCR platforms, when coupled with machine learning classifiers such as random forests or support vector machines, enable robust discrimination between healthy, intermediate, and dysbiotic states.^482^ Nanopore-based sequencing workflows are emerging as accurate, culture-free, and rapid approaches for community-level profiling directly from urogenital samples, providing a potential path to low-cost, point-of-care sequencing.^483^ At the same time, explainable AI models trained on high-throughput vaginal datasets are demonstrating strong performance in predicting outcomes ranging from in vitro fertilization success to systemic comorbidity risk.^484^ ^,^ ^485^

Dodani & Talhouk harmonized vaginal 16S rRNA data from five independent cohorts and developed a multi-cohort ensemble classifier for endometrial cancer detection, identifying reproducible microbial features, including *Peptoniphilus* enrichment, and achieving strong held-out performance, supporting the feasibility of vaginal microbiome profiles as minimally invasive biomarkers for distal uterine disease.^486^ Ojo et al. showed that machine-learning classifiers for symptomatic bacterial vaginosis exhibited ethnicity-dependent predictive performance and taxonomic feature importance, including lower accuracy among Black women, indicating that vaginal microbiome biomarker models require population-stratified validation before they can be generalized across clinical populations.^455^ Peng et al. profiled oral, fecal, urinary, and vaginal microbiota in cervical cancer patients with and without lymph-node metastasis and found that the strongest prediction model was based on oral rather than vaginal taxa, indicating that vaginal microbiome biomarkers must be benchmarked against other microbial compartments and clinical predictors before being prioritized for prognostic use.^487^ Together, these studies indicate that vaginal microbiome profiling shows promise but requires population-stratified validation and benchmarking against alternative sampling sites before clinical deployment.

An important future research direction is to determine whether vaginal microbiome profiles provide information that is independent of, or complementary to, conventional clinical variables and gut-derived measurements. If gut microbial signatures can stratify risk for neurologic conditions such as Alzheimer’s or Parkinson’s disease, it might be plausible that analogous predictive models could be developed using vaginal data.^453^ Because cervicovaginal swabs can be self-collected and easily integrated into gynecologic care, and new computational tools can detect even subtle shifts in community structure, previously indecipherable data may become clinically important—such as early indicators of systemic dysbiosis with neurologic implications. Although gut microbial signatures have been investigated as predictors of neurologic outcomes, comparable models based on cervicovaginal profiles have not been validated. Romano et al. performed a 4,489-sample machine-learning meta-analysis of Parkinson’s disease gut microbiomes and found that within-study classification outperformed cross-study transfer, indicating that microbiome-based neurologic prediction is feasible but cohort-sensitive and should not be extrapolated to vaginal data without direct validation.^488^ Whether vaginal data provides independent or complementary information regarding neurologic risk therefore remains an open research question. Integration into cervical screening, prenatal care, and other gynecologic workflows could provide a practical platform for testing these hypotheses at scale. At present, however, cervicovaginal profiling should be considered a candidate complementary biomarker platform rather than a validated surrogate for gut or neurologic states.

## 5. Conclusion

Current evidence supports a systems-level framework in which shared host regulators, selected cross-site carriage, and indirect metabolite and immune pathways create measurable coupling between gut and cervicovaginal niches. Whether this coupling is sufficiently stable and informative for cross-compartment prediction remains unresolved. The field is now positioned to test this question directly through paired longitudinal multi-omics, strain-resolved analysis, mechanistic modeling, and external validation, transforming the proposed axis from a conceptual framework into a falsifiable predictive research program.

The gut and vaginal microbiomes are best understood not as isolated niches, but as interconnected ecosystems within a unified immunometabolic network. Emerging evidence indicates that cervicovaginal communities, shaped by systemic endocrine, immune, and metabolic cues, may serve as accessible proxies for gut and whole-body states, including those relevant to neurologic health. At the same time, patterns of divergence, where gut and vaginal profiles fail to align, underscore the diagnostic importance of asymmetric dysbiosis, which may signal local pathology, treatment effects, or previously unrecognized inflammatory drivers.

These insights point directly to translational opportunities. Advances in sequencing, qPCR panels, and machine learning classifiers now make it feasible to monitor the vaginal microbiome at scale, while probiotics, prebiotics, and next-generation microbiota-based therapeutics show emerging potential for benefits across compartments. The next frontier lies in integrating multi-omic datasets across sites and life stages, validating predictive markers in large and diverse cohorts, and designing interventions that deliberately harness cross-biome coherence.

By reframing the cervicovaginal microbiome as both a sentinel biomarker and a therapeutic target, the field moves closer to a systems-level model of multifactorial disease. This approach has the potential not only to advance women’s health but also to expand the clinical utility of microbiome science into domains as far-reaching as neurology, endocrinology, and personalized medicine.

## Data Availability

There is no supporting data associated with this article.

## Statement of significance

The gut and cervicovaginal microbiomes are emerging as interconnected components of a shared endocrine, immune, metabolic, and inflammatory network with major relevance to women’s health and systemic disease. This review advances the cervicovaginal-gut microbiota axis as a powerful framework for understanding how microbial states across body sites shape physiology, disease susceptibility, and therapeutic response. Advances in sequencing, multi-omics, computational modeling, and diagnostic profiling now make it possible to interrogate this axis with unprecedented precision. By synthesizing current evidence and emerging technologies, this review positions the cervicovaginal-gut axis as an important frontier for biomarker discovery, disease prediction, and next-generation microbiome-informed therapeutics.

## Search strategy and selection criteria

Data for this Review were identified by searches of MEDLINE, PubMed, and references from relevant articles using various phrases, including the search criteria (terms): “Gut” or “Vaginal” or “Cervicovaginal” + “microbiome” or “dysbiosis” and “dysbiosis” + ”human microbiome” or ”in disease states” or “during pregnancy” or “Gynecological states” and “microbiome” + “immune” or “metabolic” or “immunochemical” or “neuroimmune” or “Immunometabolic” + ” effect” or “control” or “regulation”.

## Abbreviations

ACTH: Adrenocorticotropic hormone
AhR: Aryl hydrocarbon receptor
BV: Bacterial vaginosis
CSTs: Community state type(s)
FBA: Flux balance analysis
GDM: Gestational diabetes mellitus
HRT: Hormone replacement therapy
HIV: Human Immunodeficiency Virus
HPV: Human papillomavirus
HPA axis: Hypothalamic–pituitary–adrenal axis
ILA: Indole-3-lactate
IBD: Inflammatory bowel disease
IECs: Intestinal epithelial cell(s)
IBS: Irritable bowel syndrome
MBTs: Microbiota-based therapeutic(s)
NK: Natural killer cells
PCOS: Polycystic ovary syndrome
PE: Preeclampsia
SPE: Severe preeclampsia
STI: Sexually transmitted infection
SCFAs: Short-chain fatty acid(s)
TRMs: Tissue resident memory cells
VMT: Vaginal microbiota transplantation
